# Microbiota dysbiosis and functional outcome in acute ischemic stroke patients

**DOI:** 10.1038/s41598-021-90463-5

**Published:** 2021-05-26

**Authors:** Yoonkyung Chang, Ho Geol Woo, Jee Hyang Jeong, Geon Ha Kim, Kee Duk Park, Tae-Jin Song

**Affiliations:** 1 Department of Neurology , Ewha Womans University Mokdong Hospital, Ewha Womans University College of Medicine, Seoul, Korea; 2 Department of Neurology, Kyung Hee University College of Medicine, Seoul, Korea; 3 Department of Neurology , Ewha Womans University Seoul Hospital, Ewha Womans University College of Medicine , 260, Gonghang-daero, Gangseo-gu, 07804 Seoul, Republic of Korea

**Keywords:** Neurology, Cerebrovascular disorders

## Abstract

Currently, few studies are reported on the composition of microbiota in stroke patients and the association with stroke prognosis. This study investigated the differing microbiota composition in stroke patients and confirmed the association of microbiota composition with poor functional outcome. Between January of 2018 and December of 2019, 198 patients with acute cerebral infarction were included in this study. For the case–control study, age and sex-matched normal healthy subjects (n = 200) were included when receiving their health screening examinations. We isolated bacterial extracellular membrane vesicles and extracted DNA from blood samples. Taxonomic assignments were performed by using the sequence reads of 16S rRNA genes following blood microbiota analysis. Statistical analysis was conducted appropriately by using Statistical Analysis System software. The mean age of the stroke patients were 63.7 ± 12.5 years, and the male sex was 58.5%. Of the total enrolled patients, poor functional outcome (modified Rankin Score ≥ 3) was noted in 19.7%. The principal component analysis of microbiota composition revealed significant differences between healthy control subjects and stroke patients. At the genus level, *Aerococcaceae(f)*, *ZB2(c), TM7-1(c),* and *Flavobacterium* were significantly increased in stroke patients compared to the healthy controls, whereas *Mucispirillum, rc4-4, Akkermansia, Clostridiales(o), Lactobacillus,* and *Stenotrophomonas* were decreased considerably. For the functional outcome after ischemic stroke, *Anaerococcus, Blautia, Dialister, Aerococcaceae(f), Propionibacterium, Microbacteriaceae(f)*, *and Rothia* were enriched in the group with good outcomes, whereas *Ruminococcaceae(f)* and *Prevotella* were enriched in the group with poor outcome. There was apparent dysbiosis of blood microbiota in patients with acute ischemic stroke compared to healthy people. *Ruminococcaceae(f)* and *Prevotella* were elevated in stroke patients with poor functional outcome.

## Introduction

Stroke refers to damage caused by blocked or diminished blood flow in specific brain regions. It can cause permanent neurologic sequelae and death^1^. Stroke is a global health problem and a major cause of economic and social burden worldwide^2,3^. Inflammatory and immune reactions occur in ischemic brain tissues after stroke^4^, which aggravates pre-existing neurologic symptoms and leads to a poor prognosis^5^. Infections and systemic inflammatory response have a significant impact on mortality after stroke^6^.

Approximately 50% of stroke patients have gastrointestinal complications such as gastrointestinal bleeding, constipation, diarrhea, and dysphagia^7^, of which are associated with neurological deterioration, poor prognosis, and mortality^8^. Interestingly, microbiota are closely related to gastrointestinal complications^9^. Previous studies suggested that changes in the composition or imbalance of microbiota after a stroke may adversely affect stroke outcome by regulating pro-inflammatory mediators or inducing stress reactions at the injured site^10,11^. However, few studies have reported on the composition of the microbiota in stroke patients and the association between this microbiota and stroke prognosis.

Meanwhile, extracellular vesicles (EVs) are one way to transfer information from eukaryotic cells, consisting of various substances such as DNA, RNA, proteins, or lipids^12,13^. Bacteria in our body also generate EVs, which are small in size, unlike EVs from human cells^14^. Various isolation methods are currently being used, including ultracentrifugation, microfiltration, and gel filtration^15^. Bacteria-derived EVs are important signaling pathways between the microbiome and host^16–18^. Through this pathway, the microbiome transmits information to the host and affects the occurrence of various diseases, including the gut-brain axis^19–21^. To date, there are few studies analyzing microbiota composition with blood bacteria-derived EVs in stroke patients.

We hypothesized that the microbiota composition is different between acute ischemic stroke patients and non-stroke patients, and the microbiota composition is associated with the poor functional outcome after stroke. This study investigated the difference in microbiota composition in patients with stroke in a case–control study and confirmed the association of microbiota composition with poor functional outcome in acute ischemic stroke by analyzing bacteria-derived EVs in the blood sample.

## Results

### Comparison of microbiota composition between stroke patients and healthy controls

**Figure 1:**
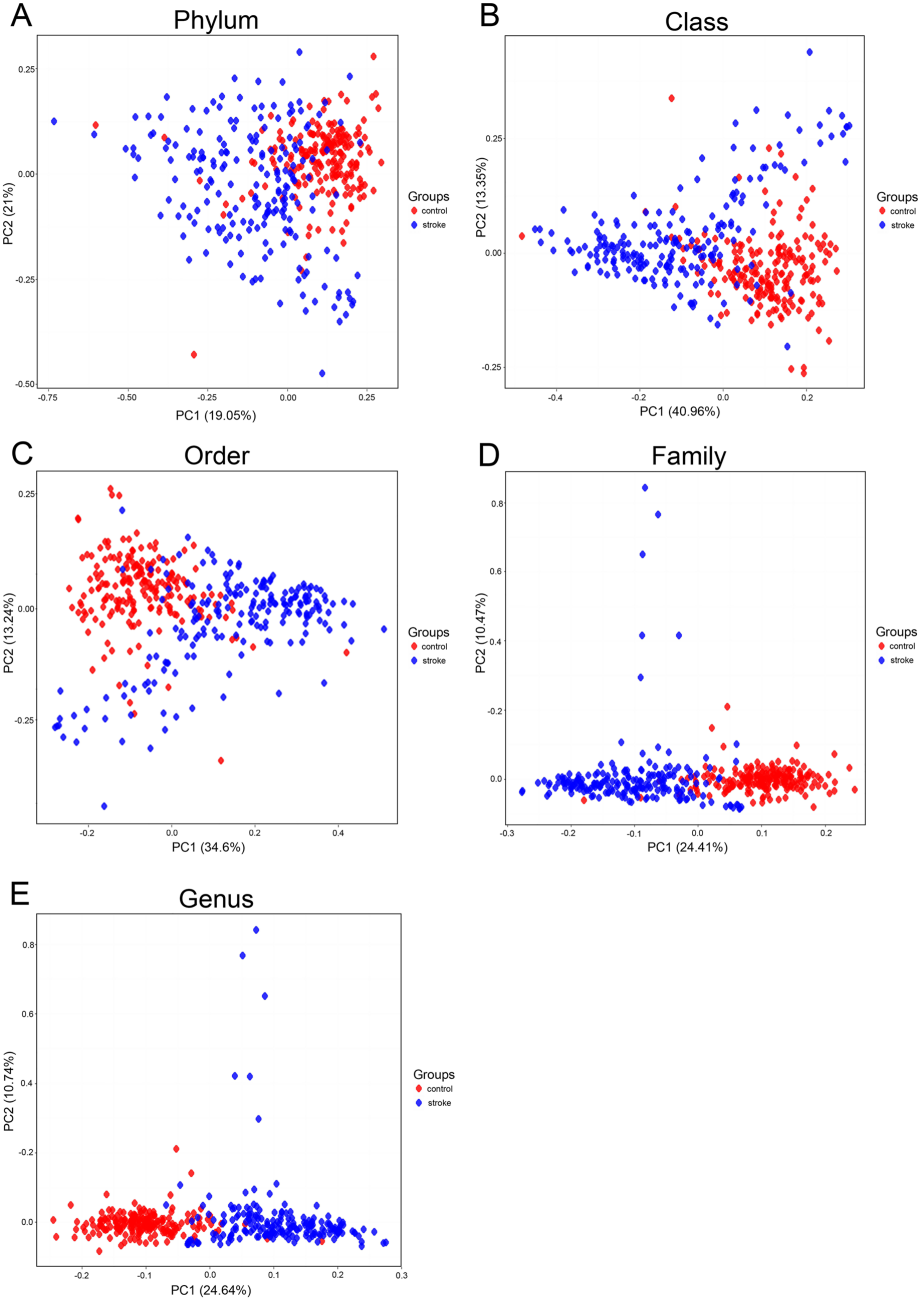
A plot of principal component analysis among patients with stroke and control groups. The relative abundance of operational taxonomic units (OTUs) accounting for > 0.1% of the total bacterial community are shown at the phylum (**A**), class (**B**), order (**C**), family (**D**), and genus (**E**) levels.

There were no differences based on age and sex between the healthy control subjects and the stroke patients (age: 63.5 ± 12.5 vs. 63.7 ± 12.5, p = 0.888; male sex: 58.6% vs. 58.5%, p = 0.906). The principal component analysis for microbiota composition revealed substantial differences between healthy control subjects and stroke patients (Fig. 1). The demographics of stroke patients are shown in Table 1.

**Table 1:** Demographics of the acute ischemic stroke patients and comparison of clinical and brain image findings according to clinical outcome at three months after index stroke.

	Total	mRS < 3 (n = 159)	mRS ≥ 3 (n = 39)	*p* value
**Demographics**				
Sex, male	116 (58.5)	98 (61.6)	18 (46.1)	0.102
Age, years	63.7 ± 12.5	62.8 ± 11.8	67.5 ± 14.6	0.034
Body mass index, kg/m^2^	24.0 ± 3.5	24.1 ± 3.4	23.5 ± 3.9	0.345
**Risk factors**				
Hypertension	115 (58.0)	91 (57.2)	24 (61.5)	0.718
Diabetes mellitus	85 (42.9)	66 (41.5)	19 (48.7)	0.472
Hypercholesterolemia	56 (28.2)	46 (28.9)	10 (25.6)	0.843
Coronary artery disease	41 (20.7)	29 (18.2)	12 (30.8)	0.120
Smoking	76 (38.3)	58 (36.5)	18 (46.2)	0.276
Alcohol intake	58 (29.2)	43 (27.0)	15 (38.5)	0.173
**Prior medication**				
Antithrombotics	44 (22.2)	35 (22.0)	9 (23.1)	1.000
Statins	46 (23.2)	41 (25.8)	5 (12.8)	0.094
NIHSS	4 [2–6]	3 [1–3]	10 [7–16]	0.001
Cerebral atherosclerosis	88 (44.4)	63 (39.6)	25 (64.1)	0.006
High-grade white matter hyperintensities	41 (20.7)	31 (19.5)	10 (25.6)	0.396
**Stroke subtype**				0.055
Cardioembolism	31 (15.6)	22 (13.8)	9 (23.1)	
Large artery atherosclerosis	78 (39.4)	59 (37.1)	19 (48.7)	
Small vessel occlusion	99 (50.0)	78 (49.1)	11 (28.2)	
**Blood laboratory findings**				
Fasting glucose, mg/dL	117.0 ± 44.3	116.2 ± 44.5	120.2 ± 43.5	0.612
HbA1c, %	6.6 ± 1.5	6.5 ± 1.5	6.9 ± 1.6	0.243
Triglyceride, mg/dL	122.8 ± 100.1	126.1 ± 107.7	109.1 ± 59.0	0.343
Total cholesterol, mg/dL	177.1 ± 38.5	175.5 ± 38.5	183.4 ± 38.2	0.253
Low density lipoprotein, mg/dL	115.9 ± 36.7	114.7 ± 36.2	120.9 ± 38.9	0.345
White blood cell count, × 10^3^	7.4 ± 2.5	7.0 ± 2.3	8.8 ± 2.9	0.001
Hemoglobin, mg/dL	13.4 ± 1.8	13.5 ± 1.8	13.2 ± 1.7	0.327
Creatinine, mg/dL	1.0 ± 0.9	0.9 ± 0.6	1.2 ± 1.4	0.222
Albumin, mg/dL	3.7 ± 0.3	3.7 ± 0.3	3.6 ± 0.4	0.082
Uric acid, mg/dL	4.8 ± 1.6	4.8 ± 1.3	5.1 ± 2.4	0.422
C-reactive protein, mg/L	0.7 ± 1.7	0.6 ± 1.4	2.2 ± 5.1	0.018

Data are shown as n (%), mean ± standard deviation, or median [interquartile range].

*mRS* modified Rankin scale, *NIHSS* National Institutes of Health Stroke Scale.

**Figure 2:**
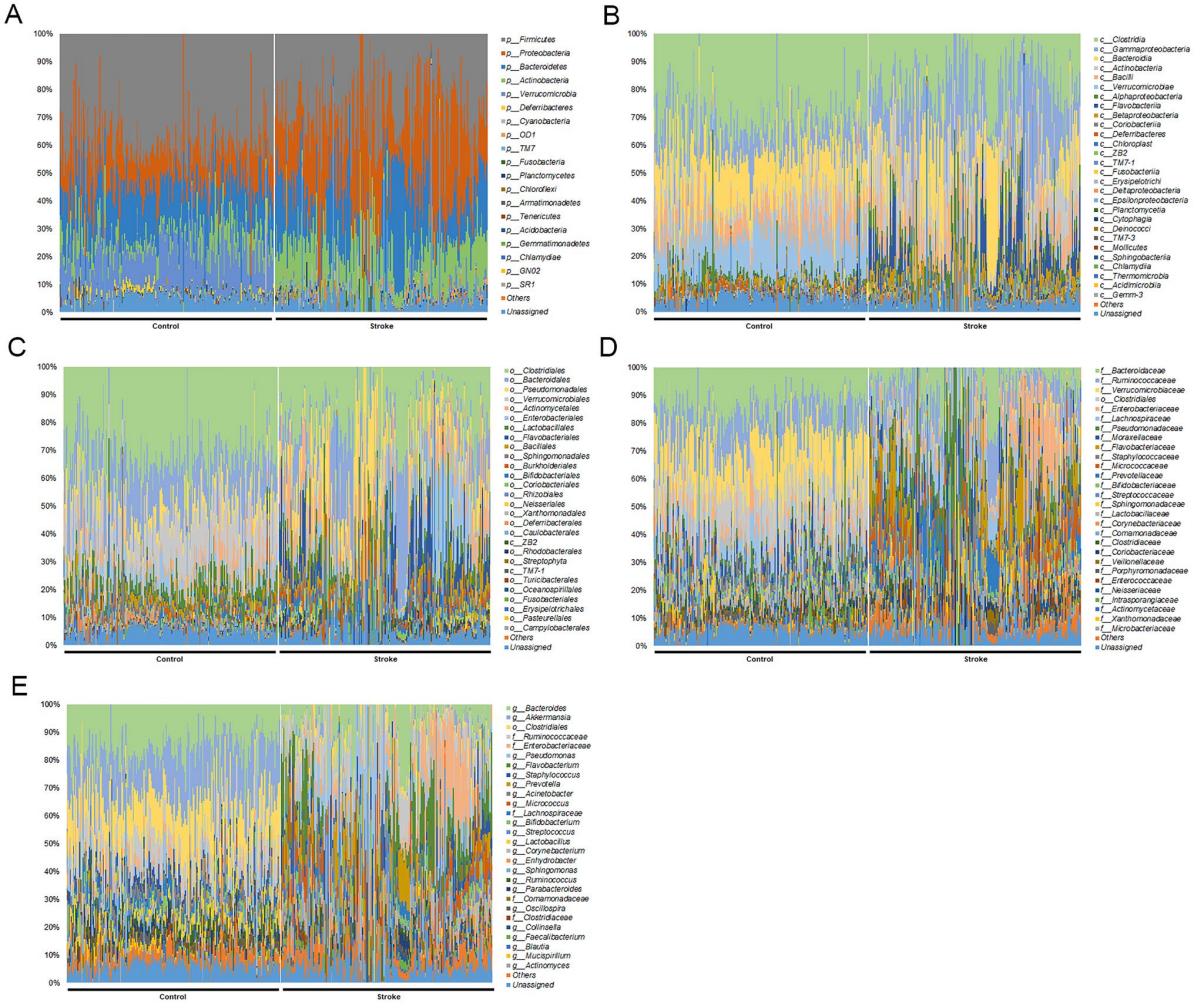
Composition of microbiota among patients with stroke and control groups. The relative abundance of operational taxonomic units (OTUs) accounting for > 0.1% of the total bacterial community are shown at the phylum (**A**), class (**B**), order (**C**), family (**D**), and genus (**E**) levels.

At the phylum level, the composition of the blood microbiota was frequently in the order of *Firmicutes, Bacteroidetes, Proteobacteria, Verrucomicrobia,* and *Actinobacteria* (which accounted for 92% of the total) in the control group. Among those, *Verrucomicrobia, Firmicutes,* and *Deferribacteres* were significantly higher in healthy controls than in stroke patients. In acute stroke patients, *Proteobacteria, Firmicutes, Bacteroidetes, Actinobacteria,* and *Verrucomicrobia* accounted for 90% of the total. *Actinobacteria, Proteobacteria, OD1,* and *TM7* were significantly higher in stroke patients than in the control group (Fig. 2a). Further comparative analysis regarding class, order, and family is demonstrated in Fig. 2b–d and Supplementary Table 1. At the genus level, *Akkermansia, Bacteroides, Lactobacillus, Ruminococcus,* and *Oscillospira* were frequently found in both groups. When comparing the two groups, *Enterobacteriaceae(f)* (p < 0.001)*, Pseudomonas* (p < 0.001)*, Flavobacterium* (p < 0.001)*, Staphylococcus* (p = 0.038), *Prevotella* (p < 0.001)*, Micrococcus* (p < 0.001)*, Corynebacterium* (p < 0.001)*, Enhydrobacter* (p < 0.001)*, Comamonadaceae(f)* (p < 0.001)*, Collinsella* (p < 0.001)*, Faecalibacterium* (p < 0.001)*, Blautia* (p = 0.021), *Anaerococcus* (p < 0.001)*, Finegoldia* (p < 0.001)*, Dialister* (p < 0.001)*, Neisseriaceae(f)* (p < 0.001)*, ZB2(c)* (p < 0.001)*, Aerococcaceae(f)* (p < 0.001), and *TM7-1(c)* (p < 0.001) were increased in stroke patients, while *Bacteroides* (p < 0.001)*, Akkermansia* (p < 0.001)*, Clostridiales(o)* (p < 0.001)*, Ruminococcaceae(f)* (p = 0.006), *Lachnospiraceae(f)* (p = 0.001)*, Lactobacillus* (p < 0.001)*, Ruminococcus* (p < 0.001)*, Parabacteroides* (p = 0.004)*, [Ruminococcus]* (p < 0.001)*, Oscillospira* (p < 0.001)*, Mucispirillum* (p < 0.001)*, Actinomyces* (p < 0.001)*, Klebsiella* (p < 0.001)*, Stenotrophomonas* (p < 0.001), and *rc4-4* (p < 0.001) were decreased in stroke patients. Of those, *Aerococcaceae(f)*, *ZB2(c), TM7-1(c),* and *Flavobacterium* were drastically increased, with 448.4-fold, 108.7-fold, 73.5-fold, and 68.6-fold elevations, respectively, whereas *Mucispirillum, rc4-4, Akkermansia, Clostridiales(o), Lactobacillus,* and *Stenotrophomonas* decreased drastically, with 0.01-fold, 0.06-fold, 0.08-fold, 0.16-fold, 0.17-fold, and 0.20-fold reductions, respectively (Table 2, Fig. 2e).

**Table 2:** Difference in microbiome composition between stroke patients and healthy controls (Genus level).

Taxon	Occupancy		Fold change
	Healthy control	Stroke	control vs stroke
*Bacteroides*	0.13819 ± 0.0042	0.06164 ± 0.0051	0.4↓
*Akkermansia*	0.14117 ± 0.0045	0.01080 ± 0.0016	0.1↓
*Clostridiales(o)*	0.12330 ± 0.0039	0.01943 ± 0.0018	0.2↓
*Ruminococcaceae(f)*	0.06609 ± 0.0027	0.05415 ± 0.0035	0.8↓
*Enterobacteriaceae(f)*	0.02437 ± 0.0016	0.07085 ± 0.0070	2.9↑
*Pseudomonas*	0.02256 ± 0.0041	0.05737 ± 0.0048	2.5↑
*Flavobacterium*	0.00087 ± 0.0003	0.05982 ± 0.0055	68.6↑
*Staphylococcus*	0.02129 ± 0.0016	0.02957 ± 0.0036	1.4↑
*Prevotella*	0.00686 ± 0.0015	0.03713 ± 0.0036	5.4↑
*Micrococcus*	0.00197 ± 0.0003	0.03889 ± 0.0032	19.8↑
*Lachnospiraceae(f)*	0.01973 ± 0.0016	0.01421 ± 0.0011	0.7↓
*Lactobacillus*	0.02594 ± 0.0009	0.00436 ± 0.0013	0.2↓
*Corynebacterium*	0.00770 ± 0.0004	0.02217 ± 0.0024	2.9↑
*Enhydrobacter*	0.00259 ± 0.0031	0.02457 ± 0.0024	9.5↑
*Ruminococcus*	0.01811 ± 0.0013	0.00702 ± 0.0009	0.4↓
*Parabacteroides*	0.01172 ± 0.0009	0.00705 ± 0.0009	0.6↓
*Comamonadaceae(f)*	0.00709 ± 0.0013	0.01132 ± 0.0017	1.6↑
*[Ruminococcus]*	0.01370 ± 0.0008	0.00453 ± 0.0006	0.3↓
*Oscillospira*	0.01232 ± 0.0006	0.00413 ± 0.0006	0.3↓
*Collinsella*	0.00310 ± 0.0004	0.00995 ± 0.0014	3.2↑
*Faecalibacterium*	0.00283 ± 0.0019	0.00961 ± 0.0010	3.4↑
*Blautia*	0.00384 ± 0.0007	0.00639 ± 0.0009	1.7↑
*Lachnospiraceae(f)*	0.00629 ± 0.0010	0.00360 ± 0.0005	0.6↓
*Mucispirillum*	0.00970 ± 0.0011	0.00013 ± 0.0001	0.0↓
*Actinomyces*	0.00709 ± 0.0004	0.00257 ± 0.0005	0.4↓
*Anaerococcus*	0.00101 ± 0.0002	0.00756 ± 0.0017	7.5↑
*Finegoldia*	0.00043 ± 0.0004	0.00810 ± 0.0018	18.9↑
*Dialister*	0.00215 ± 0.0005	0.00604 ± 0.0009	2.8↑
*Klebsiella*	0.00571 ± 0.0008	0.00215 ± 0.0004	0.4↓
*Stenotrophomonas*	0.00599 ± 0.0001	0.00118 ± 0.0005	0.2↓
*Neisseriaceae(f)*	0.00049 ± 0.0000	0.00662 ± 0.0009	13.5↑
*ZB2(c)*	0.00006 ± 0.0003	0.00691 ± 0.0008	108.7↑
*rc4-4*	0.00547 ± 0.0000	0.00033 ± 0.0002	0.1↓
*Aerococcaceae(f)*	0.00001 ± 0.0000	0.00559 ± 0.0015	448.4↑
*TM7-1(c)*	0.00007 ± 0.0000	0.00522 ± 0.0009	73.5↑

### Composition of microbiota for poor functional outcome

**Figure 3:**
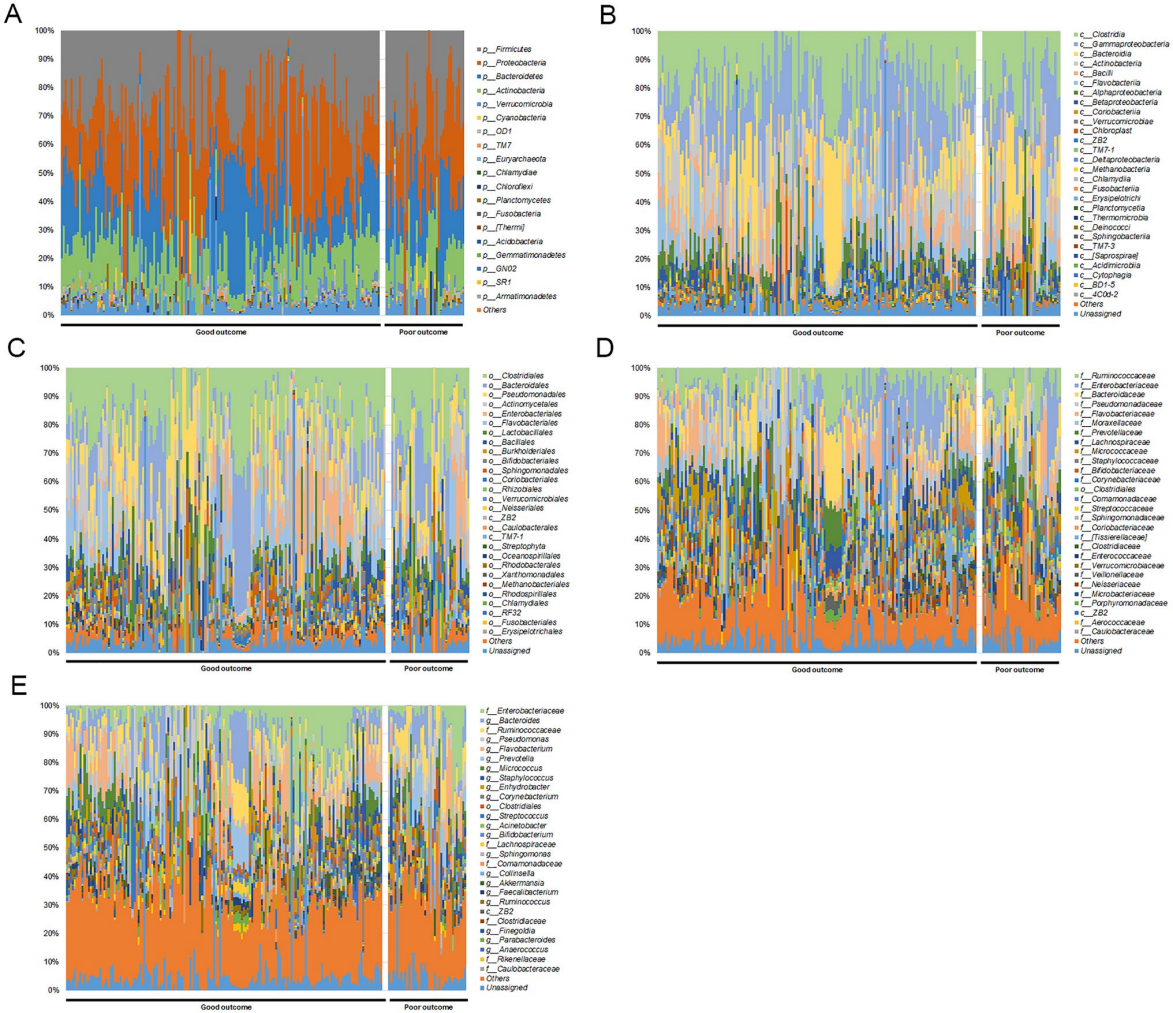
Composition of microbiota among patients with good outcomes and those with poor outcomes. The relative abundance of operational taxonomic units (OTUs) accounting for > 0.1% of the total bacterial community are shown at the phylum (**A**), class (**B**), order (**C**), family (**D**), and genus (**E**) levels.

Of the total enrolled patients, poor functional outcome was noted in 39 (19.7%). PCA analysis showed no significant differences between the two groups (Supplementary Fig. 1). *Proteobacteria, Firmicutes, Bacteroidetes*, and *Actinobacteria* were dominant phyla regardless of functional outcome. *Rhodobacterales* at the order level and *Ruminococcaceae, Prevotellaceae, [Tissierellaceae], Veillonellaceae, Microbacteriaceae, Propionibacteriaceae,* and *Rhodobacteriaceae* at the family level demonstrated significant differences between the functional outcome groups (p < 0.05) (Supplementary Table 2). At the genus level, *Ruminococcaceae(f)* (p = 0.004) and *Prevotella* (p = 0.046) in the poor outcome group were significantly higher than in the good outcome group, whereas *Anaerococcus* (p = 0.018)*, Blautia* (p = 0.039)*, Dialister* (p = 0.002)*, Aerococcaceae(f)* (p = 0.041)*, Propionibacterium* (p = 0.041)*, Microbacteriaceae(f)* (p = 0.021), and *Rothia* (p = 0.026) were significantly higher in the good outcome group than the poor outcome group (Table 3, Fig. 3).

**Table 3:** Difference in microbiome composition between functional outcome groups (Genus level).

Taxon	Occupancy			Fold change
	Good outcome	Poor outcome	p-value	good vs poor outcome
*Enterobacteriaceae(f)*	0.0732 ± 0.0077	0.0611 ± 0.0159	0.4911	0.8↓
*Bacteroides*	0.0608 ± 0.0058	0.0652 ± 0.0105	0.7302	1.1↑
*Ruminococcaceae(f)*	0.0493 ± 0.0036	0.0740 ± 0.0090	0.0045	1.5↑
*Pseudomonas*	0.0559 ± 0.0055	0.0635 ± 0.0101	0.5312	1.1↑
*Flavobacterium*	0.0637 ± 0.0062	0.0441 ± 0.0119	0.1609	0.7↓
*Prevotella*	0.0336 ± 0.0038	0.0515 ± 0.0093	0.0467	1.5↑
*Micrococcus*	0.0399 ± 0.0037	0.0349 ± 0.0065	0.5426	0.9↓
*Staphylococcus*	0.0291 ± 0.0039	0.0315 ± 0.0096	0.7945	1.1↑
*Enhydrobacter*	0.0260 ± 0.0028	0.0187 ± 0.0045	0.2385	0.7↓
*Corynebacterium*	0.0228 ± 0.0028	0.0197 ± 0.0051	0.6124	0.9↓
*Clostridiales(o)*	0.0191 ± 0.0020	0.0206 ± 0.0040	0.7371	1.1↑
*Streptococcus*	0.0168 ± 0.0028	0.0214 ± 0.0040	0.3634	1.3↑
*Acinetobacter*	0.0194 ± 0.0037	0.0177 ± 0.0046	0.7777	0.9↓
*Bifidobacterium*	0.0189 ± 0.0019	0.0161 ± 0.0032	0.5125	0.9↓
*Lachnospiraceae(f)*	0.0141 ± 0.0011	0.0147 ± 0.0029	0.8446	1.0↑
*Sphingomonas*	0.0138 ± 0.0024	0.0130 ± 0.0057	0.8919	0.9↓
*Comamonadaceae(f)*	0.0104 ± 0.0018	0.0152 ± 0.0050	0.3747	1.5↑
*Collinsella*	0.0082 ± 0.0010	0.0172 ± 0.0060	0.1511	2.1↑
*Akkermansia*	0.0111 ± 0.0019	0.0096 ± 0.0029	0.7190	0.9↓
*Faecalibacterium*	0.0100 ± 0.0012	0.0080 ± 0.0016	0.3360	0.8↓
*Ruminococcus*	0.0070 ± 0.0011	0.0070 ± 0.0021	0.9963	1.0↑
*ZB2(c)*	0.0069 ± 0.0008	0.0070 ± 0.0022	0.9438	1.0↑
*Clostridiaceae(f)*	0.0052 ± 0.0006	0.0086 ± 0.0023	0.1648	1.7↑
*Finegoldia*	0.0090 ± 0.0022	0.0043 ± 0.0014	0.0799	0.5↓
*Parabacteroides*	0.0074 ± 0.0010	0.0056 ± 0.0020	0.4270	0.8↓
*Anaerococcus*	0.0087 ± 0.0021	0.0030 ± 0.0011	0.0184	0.4↓
*Rikenellaceae(f)*	0.0052 ± 0.0008	0.0065 ± 0.0018	0.4563	1.3↑
*Caulobacteraceae(f)*	0.0029 ± 0.0008	0.0085 ± 0.0054	0.3134	3.0↑
*Neisseriaceae(f)*	0.0072 ± 0.0011	0.0041 ± 0.0014	0.0864	0.6↓
*Enterococcus*	0.0079 ± 0.0035	0.0035 ± 0.0010	0.2234	0.4↓
*Bacillu*	0.0038 ± 0.0013	0.0075 ± 0.0043	0.4232	2.0↑
*TM7-1(c)*	0.0050 ± 0.0010	0.0059 ± 0.0026	0.7594	1.2↑
*Blautia*	0.0070 ± 0.0010	0.0039 ± 0.0010	0.0399	0.6↓
*S24-7(f)*	0.0028 ± 0.0006	0.0080 ± 0.0031	0.1172	2.8↑
*[Ruminococcus]*	0.0043 ± 0.0006	0.0056 ± 0.0021	0.5542	1.3↑
*Enterococcaceae(f)*	0.0047 ± 0.0009	0.0050 ± 0.0016	0.8881	1.1↑
*Dialister*	0.0069 ± 0.0011	0.0026 ± 0.0008	0.0023	0.4↓
*Clostridiales(o)*	0.0034 ± 0.0006	0.0059 ± 0.0028	0.3843	1.8↑
*Aerococcaceae(f)*	0.0064 ± 0.0019	0.0021 ± 0.0009	0.0412	0.3↓
*Streptophyta(o)*	0.0023 ± 0.0006	0.0061 ± 0.0037	0.3253	2.6↑
*Propionibacterium*	0.0052 ± 0.0008	0.0032 ± 0.0006	0.0415	0.6↓
*Microbacteriaceae(f)*	0.0052 ± 0.0007	0.0027 ± 0.0008	0.0210	0.5↓
*Leuconostoc*	0.0014 ± 0.0009	0.0065 ± 0.0064	0.4393	4.7↑
*Actinomycetales(o)*	0.0023 ± 0.0004	0.0051 ± 0.0028	0.3270	2.2↑
*Bifidobacteriaceae(f)*	0.0020 ± 0.0010	0.0051 ± 0.0021	0.1909	2.5↑
*Rothia*	0.0051 ± 0.0012	0.0017 ± 0.0009	0.0267	0.3↓

## Discussion

In this study, dysbiosis of blood microbiota in acute ischemic stroke patients was noted compared to normal controls. At the genus level, *Aerococcaceae(f), ZB2(c), TM7-1(c),* and *Flavobacterium* were significantly increased in stroke patients, whereas *Mucispirillum, rc4-4, Akkermansia, Clostridiales(o), Lactobacillus,* and *Stenotrophomonas* were significantly decreased. Among stroke subtypes, *Lactobacillales* and *Haemophilus* were enriched in cases of large artery atherosclerosis, and *Pseudomonas* was enriched in patients with cardioembolism compared to those with small vessel occlusion. For the functional outcome after ischemic stroke, *Anaerococcus, Blautia, Dialister, Aerococcaceae(f), Propionibacterium, Microbacteriaceae(f),* and *Rothia* were significantly higher in the group with a good outcome than the group with the poor outcome. *Ruminococcaceae(f)* and *Prevotella* were significantly higher in the poor outcome group than in the good outcome group.

There is little research on microbiota composition and stroke. A small case–control study that analyzed fecal gut microbiota compositions and organic acids, serum interleukin 6 levels, and microbiota showed that Lactobacillus ruminis was higher in stroke patients than controls^22^. The authors concluded that gut dysbiosis might affect the host metabolism and inflammation, which led to stroke occurrence. In another study using fecal sample analysis, patients with stroke had higher levels of *Odoribacter, Akkermansia, Ruminococcaceae_UCG_005,* and *Victivallis* compared to controls, and *Christensenellaceae_R-7_group* had a positive correlation with clinical outcome^23^. There was a study investigating the gut microbiota composition in atherosclerotic stroke patients^24^. In this previous study, stroke patients had higher *Enterobacter, Megasphaera, Oscillibacter*, and *Desulfovibrio* and less beneficial microbes, including *Bacteroides, Prevotella,* and *Faecalibacterium*^24^. Our study differs from previous studies in microbiome composition, possibly due to differences in the design, method, and study population of the study. In common with previous studies and the results of our study, there was a significant dysbiosis of microbiota composition in stroke patients, which can lead to disruption of normal metabolism.

Compared to controls, stroke patients in our study were enriched in *Enterobacteriaceae(f), Pseudomonas, Flavobacterium, Staphylococcus, Prevotella, Micrococcus, Corynebacterium, Enhydrobacter, Comamonadaceae(f), Collinsella, Faecalibacterium, Blautia, Anaerococcus, Finegoldia, Dialister, Neisseriaceae(f), ZB2(c), Aerococcaceae(f),* and *TM7-1(c). Enterobacteriaceae, Faecalibacterium,* and *Blautia are* generally considered beneficial bacteria, while *Pseudomonas, Flavobacterium, Staphylococcus, Prevotella, Micrococcus, Corynebacterium, Enhydrobacter, Comamonadaceae, Collinsella, Anaerococcus, Finegoldia, Dialister, Neisseriaceae,* and *Aerococcaceae* are opportunistic pathogens. The association between these microbiota and stroke has not been established, except for *Prevotella*^24^. We do not know the exact mechanism and meaning of the difference in each microbiome, but this finding can be used in basic research for the microbiome-brain interaction. A detailed comparison of previous results and our study can be found in the Supplementary Table 3.

There are a few studies on the relationship between the microbiome and prognosis after acute ischemic stroke. Li et al. reported a positive correlation of *Christensenellaceae_R-7_group* and *norank_f_Ruminococcaceae* with the National Institutes of Health Stroke Scale (NIHSS) and the modified Rankin scale (mRS) score at one month and a negative correlation with *Enterobacter*^23^. Our study revealed *Ruminococcaceae(f)* and *Prevotella* were higher in the poor outcome group. *Ruminococcaceae(f)* is a butyrate-producing bacteria, and its depletion is associated with the disease. One human study reported higher stroke risk with reduced butyrate-producing bacteria, which is not in line with our study results^25^. In our study, Ruminococcaceae(f) decreased in stroke patients compared to healthy controls, whereas Ruminococcaceae(f) was higher in patients with poor outcomes. Although it is difficult to explain the reason for this discrepancy, it may be due to the small number of our patient group, difference in comparison group and different onset time after stroke. Therefore, it is difficult to conclude the benefit or harm of *Ruminococcaceae* from the results in our study.

*Prevotella* is associated with mucosal inflammation^26^, which may adversely affect recovery after stroke. Among the microbiota higher in the good outcome group, *Blautia* is associated with reduced death in graft-versus-host disease after allogeneic blood/marrow transplantation^27^. Although a mechanism is not clear, it may be that stroke prognosis results from the immune response. In a previous study, *Dialister* was enriched in stroke patients^28^, but its role in the mechanism or prognosis of a stroke is unknown. The difference between previous research and our study may be due to the difference in study design, sample size, and outcome measurement methods. Furthermore, our subjects are Koreans, and this racial difference may affect the discrepancy among the researches. Nevertheless, our research is significant as it suggests an association of microbiota composition with functional outcome in acute stroke patients.

Our study has several limitations. First, we analyzed bacteria DNA from extracellular membrane vesicles in the blood instead of small-intestinal fluid or fecal samples. Bacterial extracellular membrane vesicles contain information about DNA fragments and RNA, as well as immunomodulatory materials such as bacterial toxins and phospholipids^29,30^. As the extracellular membrane vesicles are very small, they can cross the cellular membrane of the intestine barrier and be distributed via blood throughout the body^29,30^. Therefore, blood samples could be alternative tissues for metagenome analysis of microbiota^31,32^. Second, although we enrolled patients with definite stroke subtypes, at least 20% of patients with cerebral infarction were classified as cryptogenic stroke or undetermined subtype. However, because we did not include cryptogenic stroke or undetermined subtype for the analysis, it is difficult to generalize our research results to total stroke patients. Third, patients with stroke have different risk factors and drug usage than healthy control subjects. These various factors such as accompanying risk factors or concomitant medications might have influenced the composition of blood microbiota. An additional analysis of risk factors and drug-matched control is required to confirm the difference in microbiota composition. Fourth, our study had a small sample size and was in a single center. Fifth, because our research had an association design, we could not identify causal relationships. Research on immunomodulation, such as transplanting bacteria or extracellular membrane vesicles, is necessary. Sixth, we aimed determine the impact of microbiota composition on stroke prognosis, but we could not collect samples before the stroke event. Seventh, Trimethylamine N-oxide (TMAO) is an important biomarker for the association between microbiome and cardiovascular disease^33^. Nevertheless, blood TMAO levels were not measured in our study. Lastly, a serial analysis for the blood microbiota composition could not be completed because blood sampling was done once at admission.

In conclusion, there was an apparent difference in the blood microbiota composition in patients with acute ischemic stroke compared to controls, significantly increased levels of *Aerococcaceae(f), ZB2(c), TM7-1(c),* and *Flavobacterium,* and decreased *Mucispirillum, rc4-4, Akkermansia, Clostridiales(o), Lactobacillus,* and *Stenotrophomonas*. Regarding stroke patients, *Ruminococcaceae(f)* and *Prevotella* were increased in the group with poor functional outcomes. Further research regarding the causal relationship and modification of microbiota is needed.

## Materials and methods

### Subjects

Our study had two parts. The first part compared the composition of the microbiota between normal healthy controls and acute cerebral infarction patients. The second determined whether the microbiota composition is associated with poor functional outcome in patients with acute cerebral infarction.

Between January of 2018 and December of 2019, a total of 580 acute stroke patients were admitted to our hospital within 24 h of symptom onset. Among those, 249 patients agreed to participate in the study. The subtypes of cerebral infarction were categorized by the Trial of Org 10,172 in the Acute Stroke Treatment (TOAST) classification system at discharge^34^, patients with small vessel occlusion, cardioembolism, or large artery atherosclerosis were included. Patients with undetermined cause (n = 28) was excluded in this study because this classification consists of heterogeneous etiology with different outcomes^35^. After excluding 2 patients who withdrew their consent, 219 patients were enrolled. Among those, patients with a history of any malignancy (n = 5), patients who have taken probiotics, prebiotics, or antibiotics within the last 3 months (n = 4), patients with autoimmune disease or vasculitis or on immunomodulating drugs (n = 4), patients with Parkinson’s disease (n = 2), patient with ulcerative colitis (n = 1) were excluded. (Supplementary Fig. 1) A total of 203 blood samples were analyzed for metagenomic analysis. After excluding 5 patients with flawed metagenome analysis, finally, 198 patients were included in our study. All included patients were examined by our routine stroke study protocol^36^, which includes a chest X-ray, 12-lead electrocardiography, standard blood laboratory tests after 12 h of fasting, and brain imaging studies (brain CT, MRI, CT angiography, MR angiography, or digital subtraction angiography)^37,38^.

The definition of risk factors was reported in the Supplementary Methods section of a previous study^37,39^. Neurological severity was determined with the NIHSS score at admission^40^. High-grade white matter hyperintensities (HWMHs) were a Fazekas score of ≥ 2 in deep and/or periventricular white matter^41,42^. The Kappa value was 0.92 in the presence of high-grade white matter hyperintensities. Cerebral atherosclerosis was defined as the presence of one or more vessels with more than 50% stenosis/occlusion in the intra- or extracranial cerebral arteries^43^. Stroke subtypes were grouped by the Trial of Org 10,172 in the Acute Stroke Treatment (TOAST) classification system^34^. Neurological specialists investigated three months of the mRS score as clinical outcome^44^. An mRS score of ≥ 3 at three months was considered a poor functional outcome. The Institutional Review Board approved our research (ECT 2018-11-025), and we received informed consent from all subjects and/or their care-givers.

For the case–control study, age and sex-matched healthy subjects (n = 200) were included when receiving health screening examinations at Seoul National University Hospital Healthcare System Gangnam Center (IRB No. 1502–034-647) and Inje University Haeundae Paik Hospital (IRB No. 1297992-2015-064)^45^. The control subjects had no history of stroke or any conventional vascular risk factors and clinical findings suggestive of gastrointestinal disorders. Moreover, the controls had not taken any medications, probiotics, prebiotics, or antibiotics within three months of blood sampling.

### Isolation of bacterial extracellular membrane vesicles and extraction of DNA from the blood sample

In this study, we used ultracentrifugation and microfiltration methods to isolate bacterial EVs^46–49^. Blood samples in ethylenediaminetetraacetic acid tubes were collected at admission. The serum was separated by centrifugation (1500×*g*, 15 min) at 4 °C and stored at − 70 °C until analysis. The serum samples were diluted 1:3 with 1 × PBS (pH 7.4; ML008-01, Welgene Inc., Gyeongsan, Korea) and centrifuged at 10,000×*g* for one minute at 4 °C. The supernatants were acquired and filtered with a 0.22-μm size to remove foreign particles and bacteria. Separated bacterial extracellular membrane vesicles were boiled at 100 °C for 40 min and then centrifuged at 13,000 rpm for 30 min at 4 °C. The supernatants were then acquired. Bacterial DNA was extracted from the boiled extracellular membrane vesicles with a PowerSoil DNA Isolation Kit (MO BIO Laboratories Inc., Carlsbad, CA USA) according to the manufacturer’s protocols. The DNA from the extracellular membrane vesicles in each sample was quantified with a QIAxpert system (QIAGEN, Hilden, Germany)^31,45^.

### 16S rRNA gene sequencing

The method of 16S rRNA gene sequencing was described in a previous study^32^. The hypervariable region (V3–V4) for genomic bacteria DNA was amplified according to Illumina 16S metagenomic sequencing protocols (Illumina, San Diego, CA, United States). The barcoded fusion primer sequences of 16S_V3_F (50-TCGTCGGCAGCG TCAGATGTGTATAAGAGACAG CCTACGGGNGGCWGCAG-30) and 16S_V4_R (50-GTCTCGTGGGCTCGGAGATGTG TATAAGAGACAGGACTACHVGGGTATCTAATCC-30) were utilized for amplification. To prepare the libraries, PCR products for the MiSeq System guide (Illumina) and QIAxpert (QIAGEN, Hilden, Germany) were used, respectively. After extracting and quantifying the PCR products, equimolar ratios from each mixture were analyzed and sequenced on the MiSeq platform. The data sequencing set included 1,679,505 high-quality gene sequences and a mean of 16,243 reads per sample^32^. After excluding reads with low-quality and extra-long tails trimming, the remaining representative reads were clustered into operational taxonomic units (OTUs) with a 97% similarity in sequence cut-off at the genus level^32^.

### Taxonomic assignments by sequence reads of the 16S rRNA genes following the blood microbiota analysis

Analysis of all microbiota compositions was blind to the clinical data. The methodology of microbiota analysis of the blood samples has been previously reported^32^. Based on the barcode and sequences of the primers using MiSeq (Illumina), raw pyrosequencing reads were acquired from the filtration via the sequencer^31,32^. Taxonomic assignments were performed using the profiling program MDx-Pro ver.1 (MD Healthcare, Seoul, Korea)^31,32^. High-quality sequencing reads were chosen after filtering based on the quality score (average Phred score 20) and read length (300 bp)^32^. OTUs were clustered with the sequence clustering algorithm CD-HIT^50^. The taxonomy assignment was investigated with QIIME^51^ and UCLUST against the 16S rRNA gene sequence database in GreenGenes 8.15.13 (http://qiime.org/home_static/dataFiles.html)^52^. Depending on the sequence similarity, 16S rRNA gene sequences were placed at the taxonomic levels. The composition of bacteria at each taxonomic level was plotted as a stack bar. If the case clusters could not be addressed at the genus level due to redundant sequences in the database or a lack of sequences, the taxonomic levels were addressed at higher levels, as demonstrated in parentheses^45^. Results were normalized to have a mean of 0 and standard deviation of 1^32^. Two-dimensional scatter plots with axes of the first and second principal components were generated using the Matlab 2011a^45^.

### Statistical analysis

Categorical variables were investigated by Fisher's exact test or the Chi-square test. Differences in the beta diversity of bacterial communities were tested using the non-parametric Permutational Multivariate Analysis of Variance (PERMANOVA). The clustering pattern and characteristics were analyzed by the Kruskal–Wallis test based on significant differences in the Shannon index. Statistical analysis was conducted with SAS software (version 9.3; SAS Institute, Cary, NC, United States). A p-value of less than 0.05 indicates statistical significance.

### Ethics approval

This study was approved by the institutional research ethics committee of Seoul National University Hospital Healthcare System Gangnam Center (IRB No. 1502-034-647), Inje University Haeundae Paik Hospital (IRB No. 1297992-2015-064), and Ewha Womans University Mokdong Hospital (IRB No. 2018-11-025). The procedures used in this study adhere to the tenets of the Declaration of Helsinki.

## Supplementary Information

Supplementary Information 1.

Supplementary Information 2.

## Data Availability

Data is available at 10.6084/m9.figshare.12813953.
