# Supplementary material for: Microbiota dysbiosis and functional outcome in acute ischemic stroke patients
Source: Sci Rep. 2021 May 26;11:10977. doi: 10.1038/s41598-021-90463-5 (PMC8155119; doi:10.1038/s41598-021-90463-5)
Supplement: Supplementary file 1 — Supplementary Information 1. [file 41598_2021_90463_MOESM1_ESM.doc]

**Supplementary Methods**

*Risk factors*

The definition of risk factors was described in previous studies. In brief, body mass index was estimated by dividing body weight by height (kg/m^2^). Hypertension was diagnosed when a patient had been taking anti-hypertensive medication or had a resting systolic blood pressure ≥140 mmHg or diastolic blood pressure ≥90 mmHg on repeated measurements. Diabetes mellitus was diagnosed if the patient had a fasting blood glucose level of ≥7.0 mmol/L or was being treated with oral hypoglycemic agents or insulin. Hyperlipidemia was diagnosed if the patient had low-density lipoprotein cholesterol ≥4.1 mmol/L, total cholesterol ≥6.2 mmol/L, or if the patient was treated with statins after diagnosis of hyperlipidemia. Patients were defined as smokers if they were current smokers or had stopped smoking within one year before the index stroke. Subjects whose recent mean weekly alcohol intake had regularly exceeded 300 g of ethanol were classified as heavy drinkers. The presence of coronary artery disease was determined when a patient had a history of unstable angina, myocardial infarction or angiographically confirmed coronary artery disease.^1^

**Reference**

1 Song, T. *J. et a*l. Association between Aortic Atheroma and Cerebral Small Vessel Disease in Patients with Ischemic Stroke*. J Stro*k**e** 18, 312-320, doi:10.5853/jos.2016.00171 (2016).
