# Supplementary material for: Microbiota dysbiosis and functional outcome in acute ischemic stroke patients
Source: Sci Rep. 2021 May 26;11:10977. doi: 10.1038/s41598-021-90463-5 (PMC8155119; doi:10.1038/s41598-021-90463-5)
Supplement: Supplementary file 2 — Supplementary Information 2. [file 41598_2021_90463_MOESM2_ESM.doc]

**Title**: Microbiota dysbiosis and functional outcome in acute ischemic stroke patients

**Running title**: Dysbiosis of microbiota and stroke outcome

**Authors**:

Yoonkyung Chang, MD^1^, Ho Geol Woo, MD, PhD^2^, Jee Hyang Jeong, MD, PhD^1^, Geon Ha Kim, MD, PhD^1^, Kee Duk Park, MD, PhD^1^, Tae-Jin Song, MD, PhD^3^

^1^Department of Neurology, Mokdong Hospital, Ewha Womans University College of Medicine, Seoul, Korea

^2^Department of Neurology, Kyung Hee University College of Medicine, Seoul, Korea

^3^Department of Neurology, Seoul Hospital, Ewha Womans University College of Medicine, Seoul, Korea

**Correspondence**

Tae-Jin Song, MD, PhD.

Department of Neurology, Seoul Hospital, Ewha Womans University College of Medicine, 260, Gonghang-daero, Gangseo-gu, 07804 Seoul, Republic of Korea

Tel: +82-2-6986-1672, Fax: +82-2-6986-7000; E-mail: [knstar@ewha.ac.kr](mailto:knstar@ewha.ac.kr)

ORCID: 0000-0002-9937-762X

**Supplementary Table 1.** Comparison of microbiome composition between acute stroke patients and healthy controls in phylum, class, order and family levels.

| Phylum | | |  | Class | | |  | Order | | |  | Family | | |
| --- | --- | --- | --- | --- | --- | --- | --- | --- | --- | --- | --- | --- | --- | --- |
| Taxon | Occupancy | |  | Taxon | Occupancy | |  | Taxon | Occupancy | |  | Taxon | Occupancy | |
|  | Healthy control | Stroke |  |  | Healthy control | Stroke |  |  | Healthy control | Stroke |  |  | Healthy control | Stroke |
| *Firmicutes* | 0.3974 | 0.2778 |  | *Clostridia* | 0.3167 | 0.1886 |  | *Clostridiales* | 0.3167 | 0.1879 |  | *Ruminococcaceae* | 0.1009 | 0.0752 |
|  |  |  |  |  |  |  |  |  |  |  |  | *Clostridiales(o)* | 0.1233 | 0.0194 |
|  |  |  |  |  |  |  |  |  |  |  |  | *Lachnospiraceae* | 0.0530 | 0.0438 |
|  |  |  |  |  |  |  |  |  |  |  |  | *Clostridiaceae* | 0.0124 | 0.0108 |
|  |  |  |  |  |  |  |  |  |  |  |  | *Veillonellaceae* | 0.0115 | 0.0103 |
|  |  |  |  |  |  |  |  |  |  |  |  | *[Tissierellaceae]* | 0.0020 | 0.0192 |
|  |  |  |  |  |  |  |  |  |  |  |  | *Peptococcaceae* | 0.0056 | 0.0008 |
|  |  |  |  | *Bacilli* | 0.0780 | 0.0870 |  | *Bacillales* | 0.0241 | 0.0382 |  | *Staphylococcaceae* | 0.0214 | 0.0296 |
|  |  |  |  |  |  |  |  |  |  |  |  | *Bacillaceae* | 0.0018 | 0.0052 |
|  |  |  |  |  |  |  |  | *Lactobacillales* | 0.0491 | 0.0473 |  | *Streptococcaceae* | 0.0168 | 0.0185 |
|  |  |  |  |  |  |  |  |  |  |  |  | *Lactobacillaceae* | 0.0261 | 0.0044 |
|  |  |  |  |  |  |  |  |  |  |  |  | *Enterococcaceae* | 0.0037 | 0.0122 |
|  |  |  |  |  |  |  |  |  |  |  |  | *Aerococcaceae* | 0.0004 | 0.0070 |
| *Proteobacteria* | 0.1479 | 0.2822 |  | *Alphaproteobacteria* | 0.0243 | 0.0492 |  | *Sphingomonadales* | 0.0152 | 0.0228 |  | *Sphingomonadaceae* | 0.0148 | 0.0184 |
|  |  |  |  |  |  |  |  | *Rhizobiales* | 0.0024 | 0.0108 |  |  |  |  |
|  |  |  |  |  |  |  |  | *Caulobacterales* | 0.0033 | 0.0047 |  |  |  |  |
|  |  |  |  |  |  |  |  | *Rhodobacterales* | 0.0015 | 0.0049 |  |  |  |  |
|  |  |  |  | *Betaproteobacteria* | 0.0159 | 0.0352 |  | *Burkholderiales* | 0.0126 | 0.0246 |  | *Comamonadaceae* | 0.0088 | 0.0181 |
|  |  |  |  |  |  |  |  | Neisseriales | 0.0018 | 0.0097 |  | Neisseriaceae | 0.0018 | 0.0097 |
|  |  |  |  | Gammaproteobacteria | 0.1028 | 0.1944 |  | Pseudomonadales | 0.0518 | 0.1050 |  | Pseudomonadaceae | 0.0240 | 0.0582 |
|  |  |  |  |  |  |  |  |  |  |  |  | Moraxellaceae | 0.0278 | 0.0468 |
|  |  |  |  |  |  |  |  | Enterobacteriales | 0.0389 | 0.0785 |  | Enterobacteriaceae | 0.0389 | 0.0785 |
|  |  |  |  |  |  |  |  | Xanthomonadales | 0.0076 | 0.0039 |  | Xanthomonadaceae | 0.0075 | 0.0036 |
| Bacteroidetes | 0.1701 | 0.1882 |  | Bacteroidia | 0.1623 | 0.1199 |  | Bacteroidales | 0.1623 | 0.1199 |  | Bacteroidaceae | 0.1382 | 0.0616 |
|  |  |  |  |  |  |  |  |  |  |  |  | Prevotellaceae | 0.0069 | 0.0371 |
|  |  |  |  |  |  |  |  |  |  |  |  | Porphyromonadaceae | 0.0126 | 0.0077 |
|  |  |  |  |  |  |  |  |  |  |  |  | Rikenellaceae | 0.0013 | 0.0054 |
|  |  |  |  | Flavobacteriia | 0.0048 | 0.0647 |  | Flavobacteriales | 0.0048 | 0.0647 |  | Flavobacteriaceae | 0.0023 | 0.0600 |
| Actinobacteria | 0.0626 | 0.1381 |  | Actinobacteria | 0.0543 | 0.1218 |  | Actinomycetales | 0.0389 | 0.1004 |  | Micrococcaceae | 0.0061 | 0.0446 |
|  |  |  |  |  |  |  |  |  |  |  |  | Corynebacteriaceae | 0.0077 | 0.0222 |
|  |  |  |  |  |  |  |  |  |  |  |  | Intrasporangiaceae | 0.0075 | 0.0038 |
|  |  |  |  |  |  |  |  |  |  |  |  | Actinomycetaceae | 0.0079 | 0.0032 |
|  |  |  |  |  |  |  |  |  |  |  |  | Microbacteriaceae | 0.0004 | 0.0100 |
|  |  |  |  |  |  |  |  | Bifidobacteriales | 0.0154 | 0.0215 |  | Bifidobacteriaceae | 0.0154 | 0.0215 |
|  |  |  |  | Coriobacteriia | 0.0081 | 0.0147 |  | Coriobacteriales | 0.0081 | 0.0147 |  | Coriobacteriaceae | 0.0081 | 0.0147 |
| Verrucomicrobia | 0.1415 | 0.0118 |  | Verrucomicrobiae | 0.1412 | 0.0112 |  | Verrucomicrobiales | 0.1412 | 0.0112 |  | Verrucomicrobiaceae | 0.1412 | 0.0112 |
| Deferribacteres | 0.0097 | 0.0001 |  | Deferribacteres | 0.0097 | 0.0001 |  | Deferribacterales | 0.0097 | 0.0001 |  | Deferribacteraceae | 0.0097 | 0.0001 |
| *Cyanobacteria* | 0.0029 | 0.0064 |  | *Chloroplast* | 0.0027 | 0.0053 |  | *Streptophyta* | 0.0026 | 0.0031 |  |  |  |  |
| *OD1* | 0.0001 | 0.0081 |  | *ZB2* | 0.0001 | 0.0069 |  | *ZB2(c)* | 0.0001 | 0.0069 |  | *ZB2(c)* | 0.0001 | 0.0069 |
| *TM7* | 0.0010 | 0.0070 |  | *TM7-1* | 0.0001 | 0.0052 |  | *TM7-1(c)* | 0.0001 | 0.0052 |  | *TM7-1(c)* | 0.0001 | 0.0052 |
| ***Summation*** | **0.9332** | **0.9198** |  |  | **0.9210** | **0.9043** |  |  | **0.9083** | **0.8860** |  |  | **0.8677** | **0.8051** |

**Supplementary Table 2.** Comparison of microbiome composition between functional outcome groups in phylum, class, order and family levels.

| Phylum | | |  | Class | | |  | Order | | |  | Family | | |
| --- | --- | --- | --- | --- | --- | --- | --- | --- | --- | --- | --- | --- | --- | --- |
| Taxon | Occupancy | |  | Taxon | Occupancy | |  | Taxon | Occupancy | |  | Taxon | Occupancy | |
|  | Good outcome | Poor outcome |  |  | Good outcome | Poor outcome |  |  | Good outcome | Poor outcome |  |  | Good outcome | Poor outcome |
| Firmicutes | 0.2755 | 0.2874 |  | Clostridia | 0.1877 | 0.1922 |  | Clostridiales | 0.1869 | 0.1922 |  | Ruminococcaceae | 0.0709 | 0.0927 |
|  |  |  |  |  |  |  |  |  |  |  |  | Lachnospiraceae | 0.0449 | 0.0394 |
|  |  |  |  |  |  |  |  |  |  |  |  | Clostridiales(o) | 0.0191 | 0.0206 |
|  |  |  |  |  |  |  |  |  |  |  |  | [Tissierellaceae] | 0.0213 | 0.0109 |
|  |  |  |  |  |  |  |  |  |  |  |  | Clostridiaceae | 0.0102 | 0.0131 |
|  |  |  |  |  |  |  |  |  |  |  |  | Veillonellaceae | 0.0114 | 0.0060 |
|  |  |  |  | Bacilli | 0.0857 | 0.0922 |  | Lactobacillales | 0.0470 | 0.0488 |  | Streptococcaceae | 0.0178 | 0.0214 |
|  |  |  |  |  |  |  |  |  |  |  |  | Enterococcaceae | 0.0127 | 0.0101 |
|  |  |  |  |  |  |  |  |  |  |  |  | Aerococcaceae | 0.0071 | 0.0067 |
|  |  |  |  |  |  |  |  |  |  |  |  | Leuconostocaceae | 0.0027 | 0.0069 |
|  |  |  |  |  |  |  |  |  |  |  |  | Lactobacillaceae | 0.0050 | 0.0018 |
|  |  |  |  |  |  |  |  | Bacillales | 0.0373 | 0.0419 |  | Staphylococcaceae | 0.0291 | 0.0315 |
|  |  |  |  |  |  |  |  |  |  |  |  | Bacillaceae | 0.0045 | 0.0081 |
| Proteobacteria | 0.2834 | 0.2775 |  | Gammaproteobacteria | 0.1978 | 0.1806 |  | Pseudomonadales | 0.1057 | 0.1019 |  | Pseudomonadaceae | 0.0567 | 0.0642 |
|  |  |  |  |  |  |  |  |  |  |  |  | Moraxellaceae | 0.0490 | 0.0376 |
|  |  |  |  |  |  |  |  | Enterobacteriales | 0.0807 | 0.0695 |  | Enterobacteriaceae | 0.0807 | 0.0695 |
|  |  |  |  | Alphaproteobacteria | 0.0480 | 0.0540 |  | Sphingomonadales | 0.0240 | 0.0182 |  | Sphingomonadaceae | 0.0190 | 0.0161 |
|  |  |  |  |  |  |  |  | Rhizobiales | 0.0094 | 0.0162 |  | Methylobacteriaceae | 0.0028 | 0.0090 |
|  |  |  |  |  |  |  |  | Caulobacterales | 0.0034 | 0.0097 |  | Caulobacteraceae | 0.0034 | 0.0097 |
|  |  |  |  |  |  |  |  | Rhodobacterales | 0.0055 | 0.0024 |  | Rhodobacteraceae | 0.0053 | 0.0023 |
|  |  |  |  | Betaproteobacteria | 0.0345 | 0.0379 |  | Neisseriales | 0.0106 | 0.0064 |  | Neisseriaceae | 0.0106 | 0.0064 |
|  |  |  |  |  |  |  |  | Burkholderiales | 0.0230 | 0.0311 |  | Alcaligenaceae | 0.0023 | 0.0055 |
|  |  |  |  |  |  |  |  |  |  |  |  | Comamonadaceae | 0.0170 | 0.0224 |
| Bacteroidetes | 0.1879 | 0.1894 |  | Bacteroidia | 0.1148 | 0.1405 |  | Bacteroidales | 0.1148 | 0.1405 |  | Bacteroidaceae | 0.0608 | 0.0652 |
|  |  |  |  |  |  |  |  |  |  |  |  | Prevotellaceae | 0.0336 | 0.0515 |
|  |  |  |  |  |  |  |  |  |  |  |  | Porphyromonadaceae | 0.0080 | 0.0066 |
|  |  |  |  |  |  |  |  |  |  |  |  | Rikenellaceae | 0.0052 | 0.0065 |
|  |  |  |  |  |  |  |  |  |  |  |  | S24-7 | 0.0028 | 0.0080 |
|  |  |  |  | Flavobacteriia | 0.0692 | 0.0464 |  | Flavobacteriales | 0.0692 | 0.0464 |  | Flavobacteriaceae | 0.0639 | 0.0442 |
| Actinobacteria | 0.1394 | 0.1328 |  | Actinobacteria | 0.1244 | 0.1112 |  | Actinomycetales | 0.1030 | 0.0896 |  | Micrococcaceae | 0.0463 | 0.0375 |
|  |  |  |  |  |  |  |  |  |  |  |  | Corynebacteriaceae | 0.0228 | 0.0197 |
|  |  |  |  |  |  |  |  |  |  |  |  | Propionibacteriaceae | 0.0052 | 0.0032 |
|  |  |  |  |  |  |  |  |  |  |  |  | Actinomycetales(o) | 0.0023 | 0.0051 |
|  |  |  |  |  |  |  |  |  |  |  |  | Microbacteriaceae | 0.0110 | 0.0059 |
|  |  |  |  |  |  |  |  | Bifidobacteriales | 0.0214 | 0.0216 |  | Bifidobacteriaceae | 0.0214 | 0.0216 |
|  |  |  |  | Coriobacteriia | 0.0132 | 0.0206 |  | Coriobacteriales | 0.0132 | 0.0206 |  | Coriobacteriaceae | 0.0132 | 0.0206 |
| Verrucomicrobia | 0.0122 | 0.0103 |  | Verrucomicrobiae | 0.0116 | 0.0097 |  | Verrucomicrobiales | 0.0116 | 0.0097 |  | Verrucomicrobiaceae | 0.0116 | 0.0097 |
| Cyanobacteria | 0.0052 | 0.0113 |  | Chloroplast | 0.0042 | 0.0098 |  | Streptophyta | 0.0023 | 0.0061 |  | Streptophyta(o) | 0.0023 | 0.0061 |
| OD1 | 0.0081 | 0.0082 |  | ZB2 | 0.0069 | 0.0070 |  | ZB2(c) | 0.0069 | 0.0070 |  | ZB2(c) | 0.0069 | 0.0070 |
| TM7 | 0.0070 | 0.0071 |  | TM7-1 | 0.0050 | 0.0059 |  | TM7-1(c) | 0.0050 | 0.0059 |  | TM7-1(c) | 0.0050 | 0.0059 |
| ***Summation*** | **0.9186** | **0.9240** |  |  | **0.9032** | **0.9083** |  |  | **0.8811** | **0.8859** |  |  | **0.8261** | **0.8365** |

**Supplementary Table 3. Summary of research regarding microbiota composition in stroke patients.**

| **Authors** | **Results** | **Subjects** | **Kinds of sample of Microbiota** |
| --- | --- | --- | --- |
| Yamashiro, K. et al.^1^ | Higher serum interleukin 6 level and microbiota *Lactobacillus ruminis* in stroke patients | 41 ischemic stroke patients and 40 control subjects | fecal |
| Li, N. et al.^2^ | Higher levels of *Odoribacter, Akkermansia, Ruminococcaceae_UCG_005,* and *Victivallis* in stroke patients | 30 cerebral ischemic stroke patients and 30 healthy control | fecal |
| Yin, J. et al.^3^ | Higher *Enterobacter, Megasphaera, Oscillibacter,* and *Desulfovibrio* and less beneficial microbes, including *Bacteroides, Prevotella,* and *Faecalibacterium* in atherosclerotic stroke and TIA patients | 322 large‐artery atherosclerotic ischemic stroke and TIA patients and 231 controls | fecal |
| Zeng, X. et al.^4^ | Higher *Enterobacteriaceae, Veillonellaceae, Bifidobacterium,* and *Lactobacillus* in patients with higher risk of stroke | 141 participants without prior history of stroke | fecal |
| Ji, W. et al.^5^ | Higher *Escherichia, Bacteroides, Megamonas, Parabacteroides, Akkermansia, Prevotella, Faecalibacterium, Dialister, Bifidobacterium,* and *Ruminococcus* in stroke patients | 8 patients with cerebral infarction, 2 patients with transient ischemic attack, and 10 healthy volunteers | fecal |

**Reference**

1 Yamashiro, K. *et al.* Gut dysbiosis is associated with metabolism and systemic inflammation in patients with ischemic stroke. *PloS one* **12**, e0171521, doi:10.1371/journal.pone.0171521 (2017).

2 Li, N. *et al.* Change of intestinal microbiota in cerebral ischemic stroke patients. *BMC microbiology* **19**, 191, doi:10.1186/s12866-019-1552-1 (2019).

3 Yin, J. *et al.* Dysbiosis of Gut Microbiota With Reduced Trimethylamine-N-Oxide Level in Patients With Large-Artery Atherosclerotic Stroke or Transient Ischemic Attack. *Journal of the American Heart Association* **4**, doi:10.1161/jaha.115.002699 (2015).

4 Zeng, X. *et al.* Higher Risk of Stroke Is Correlated With Increased Opportunistic Pathogen Load and Reduced Levels of Butyrate-Producing Bacteria in the Gut. *Front Cell Infect Microbiol* **9**, 4, doi:10.3389/fcimb.2019.00004 (2019).

5 Ji, W. *et al.* Analysis of intestinal microbial communities of cerebral infarction and ischemia patients based on high throughput sequencing technology and glucose and lipid metabolism. *Molecular medicine reports* **16**, 5413-5417, doi:10.3892/mmr.2017.7227 (2017).


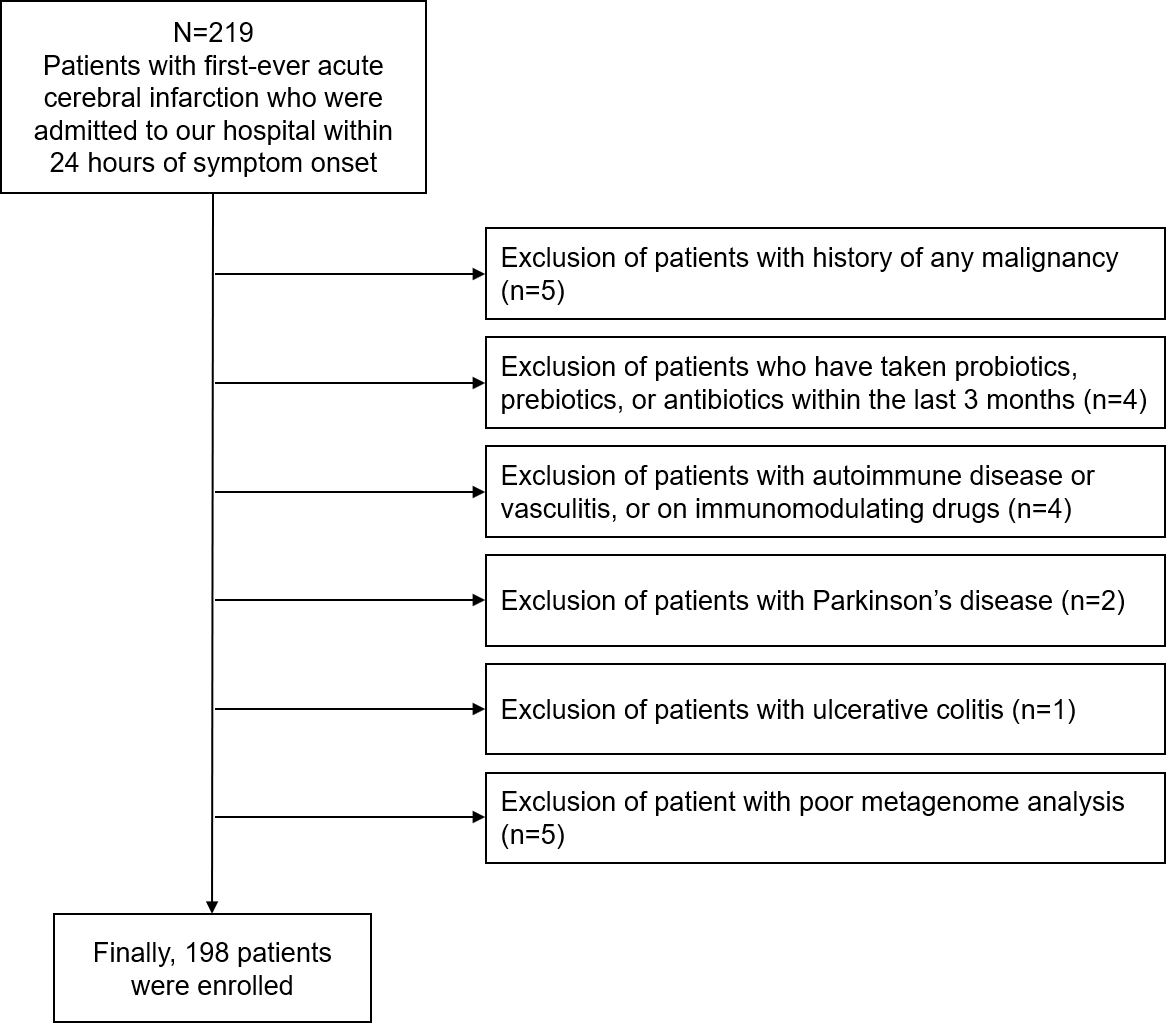


**Supplementary Figure 1.** Flowchart of the study participants

**
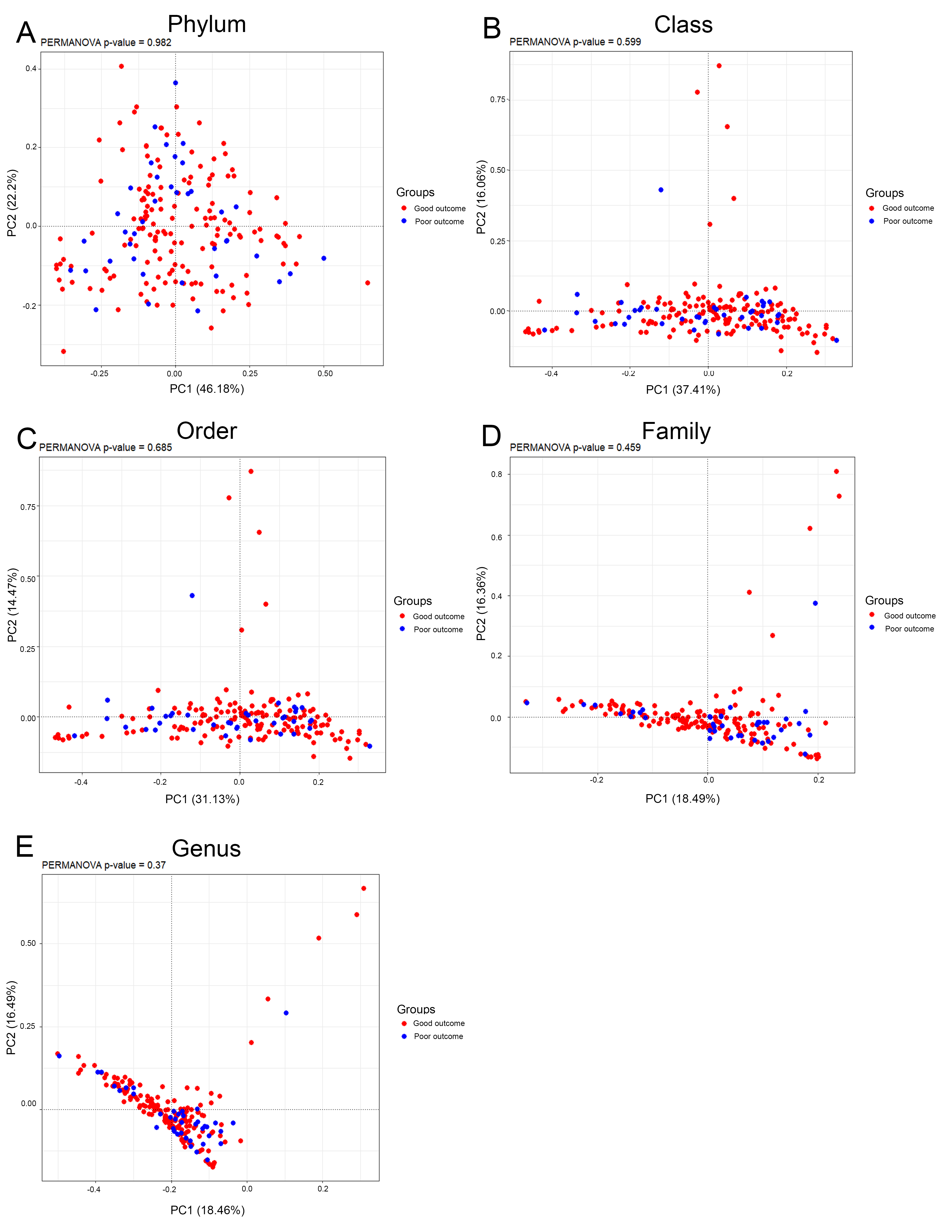
**

**Supplementary Figure 2.** The plot of a principal component analysis among patients with good outcomes and those with poor outcomes. The relative abundances of operational taxonomic units (OTUs) accounting for > 0.1% of the total bacterial community is shown at the phylum (a), class (b), order (c), family (d), and genus (e) levels.
